# Multifocal Desmoid-Type Fibromatosis: Case Series and Potential Relationship to Neuronal Spread

**DOI:** 10.7759/cureus.53771

**Published:** 2024-02-07

**Authors:** Keith M Skubitz, Shelly Marette, Paari Murugan, Bevan Yueh, Denis R Clohisy

**Affiliations:** 1 Medicine, University of Minnesota, Minneapolis, USA; 2 Radiology, University of Minnesota, Minneapolis, USA; 3 Laboratory Medicine and Pathology, University of Minnesota, Minneapolis, USA; 4 Otolaryngology, University of Minnesota, Minneapolis, USA; 5 Orthopaedic Surgery, University of Minnesota, Minneapolis, USA

**Keywords:** msh6, oncology, orthopedic oncology, beta-catenin, ctnnb1, aggressive fibromatosis, neuromuscular choristoma, desmoid tumor

## Abstract

Multifocal desmoid-type fibromatosis (DTF) is very rare and usually regional. We report three cases that initially appeared to be multifocal, but subsequent detailed imaging revealed unsuspected tracking along nerves in two cases. This neural spread is reminiscent of neuromuscular choristoma (NMC), a rare developmental lesion in which mature skeletal muscle cells, or rarely smooth muscle cells, infiltrate and enlarge peripheral nerves. NMC is frequently associated with DTF. These two cases suggest that DTF spread along nerves and appeared as distinct multifocal lesions while actually being contiguous. The third case was felt to represent true multifocal tumor development, possibly due to tumor seeding at the time of chest surgery. The relationship of DTF to NMC is discussed.

## Introduction

Desmoid tumor, also known as aggressive fibromatosis or desmoid-type fibromatosis (DTF), is a poorly circumscribed tumor composed of myofibroblast-like cells with variable collagen deposition that is felt not to metastasize [1-3]. The Wnt/beta-catenin pathway plays an important role in DTF, and mutations in APC or CTNNB1 are present in most DTF.

DTF was first reported in the abdominal wall by MacFarlane in 1832 [4]. DTF is common in patients with familial adenomatous polyposis (FAP), where a germline mutation in APC is present. Later reports described extra-abdominal DTF, which was characterized as unifocal primaries (reviewed in [5]). Sporadic DTF usually bears a mutation in CTNNB1, the gene that encodes beta-catenin [6]. Rare cases of multifocal DTF confined to one limb (reviewed in [5]) have been reported, and subsequent reports have confirmed the very rare nature of multifocal sporadic extra-abdominal DTF [5,7]; almost all cases have been regional [8-10]. Rock et al. suggested that clinical observation could be an appropriate initial treatment of DTF [11], and Lewis et al. furthered this suggestion, noting that aggressive attempts at achieving negative resection margins may result in unnecessary morbidity [12] observation is now the usual approach for most patients [1,3,13].

We report three cases that initially appeared to be multifocal sporadic DTF, two of which were later felt to reflect "subclinical" disease spread along nerves, suggesting they reflected a single atypical extensive tumor rather than true multifocal disease. The relationship of these cases to neuromuscular choristoma (NMC) [14-16] is discussed. The third case was thought to represent true multifocal tumor development, although possibly via "seeding" from previous surgery.

## Case presentation

Case 1

A 23-year-old woman developed painful multifocal DTF of both legs and gluteal muscles one month after a horse stepped on her left leg. In retrospect, she had noted decreased flexibility in both legs beginning at age 13. The leg tumor was removed and found to harbor a CTNNB1 S45F mutation. The disease recurred 10 months later, and she was treated with a variety of agents intermittently over 20 years including methotrexate/vinblastine, pegylated-liposomal doxorubicin (PLD), ifosfamide/etoposide, imatinib, and sunitinib. This case was previously reported as a case of imatinib-resistant, sunitinib-responsive DTF [17]. The sunitinib was gradually weaned, but the tumor recurred. She was then treated with ifosfamide and etoposide for three cycles with a response, but due to treatment toxicity, the treatment was changed to gemcitabine. She received six cycles of gemcitabine over three months.

**Figure 1:**
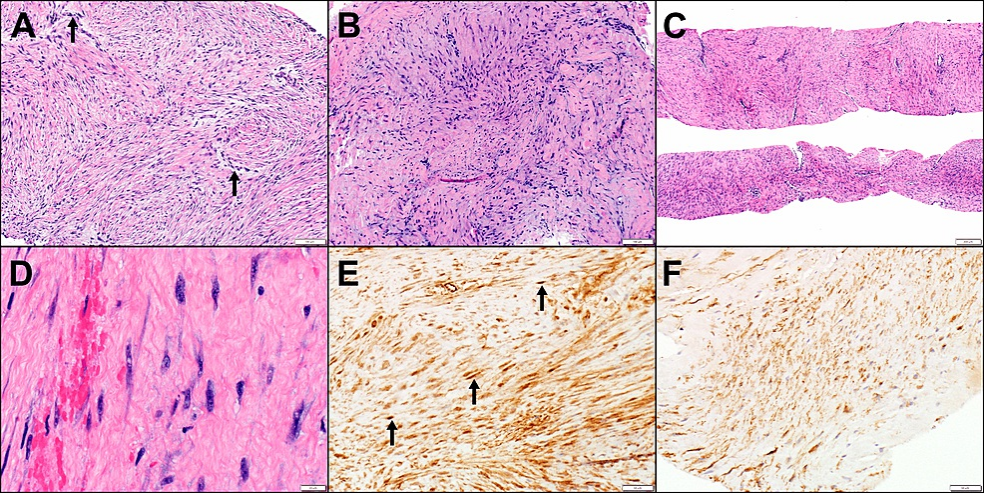
Deep DTF Vaginal mass biopsy from Case 1 showing myxoid and collagenous stroma containing low-grade spindle cells and slit-like blood vessels with perivascular edema (arrows) (H&E ×100) (A). Right thigh biopsy from Case 2 with keloidal-type collagen (right) in addition to spindle cell fascicles (H&E ×100) (B). Left chest wall biopsy from Case 3 (C-F) showing typical features of DTF with sweeping fascicles of spindle cells and interspersed compressed blood vessels (H&E ×40) (C). Higher magnification demonstrating spindled myofibroblasts with oval nuclei, multiple small nucleoli, and amphophilic cytoplasm in a collagenous background with extravasated red blood cells (left) (H&E ×400) (D). Beta-catenin immunohistochemical stain diffusely highlighting the lesional cells in a cytoplasmic pattern with scattered nuclear reactivity (arrows), typical of DTF (IHC ×200) (E). Immunohistochemical stain for smooth muscle actin with positive staining, confirming the myofibroblastic nature of the tumor cells (IHC ×200) (F) DTF: desmoid-type fibromatosis; IHC: immunohistochemistry; H&E: hematoxylin and eosin

The tumor recurred three years after the last gemcitabine. She then received 10 cycles of monthly PLD with a good response receiving a total dose of 342 mg/m2. Six years later (18 years after the original diagnosis), she developed transient hematuria, and imaging showed a mass by the uterus. The hematuria resolved. She developed several other tumors at the same time in the left posterior buttock, left chest, and right hand, all of which were tender to pressure. The hand lesion resolved. Biopsy of the left flank mass showed fat necrosis. Biopsy of the parauterine mass showed DTF (Figure 1); CTNNB1 mutation analysis showed S45F. Next-generation sequencing (NGS) by FoundationOne (Foundation Medicine, Boston, Massachusetts, United States) showed tumor mutation burden (TMB) 2 muts/Mb, CTNNB1 S45F, and microsatellite stability (MSS) stable and no other mutations.

**Figure 2:**
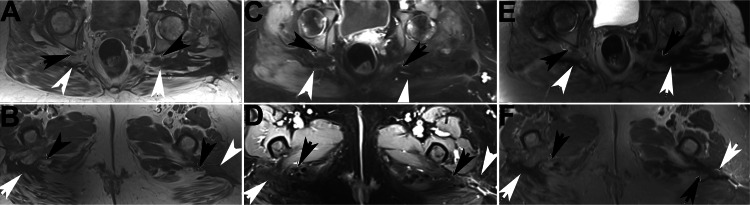
Sequential axial MR images of Case 1 Left column (A, B): T1 sequence; middle column (C, D): T1 fat saturation sequence post contrast; right column (E, F): T2 fat saturation sequence. Black arrows indicate sciatic nerve; white arrows indicate tumor

**Figure 3:**
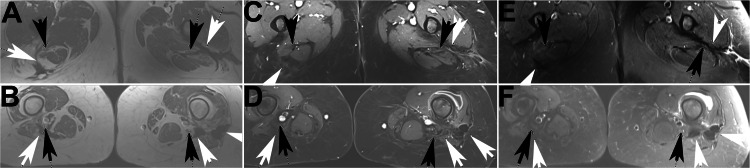
Sequential axial MR images of Case 1, continuation from Figure 2 Left column (A, B): T1 sequence; middle column (C, D): T1 fat saturation sequence post contrast; right column (E, F): T2 fat saturation sequence. Black arrows indicate sciatic nerve; white arrows indicate tumor

Detailed imaging showed the DTF in both legs to be part of a single huge DTF lesion that crossed the midline in the pelvis that was felt to track along nerves (Figure 2 and Figure 3). 

It was concluded that what was originally felt to be multifocal DTF was in fact one extensive DTF lesion involving both legs and crossing at the pelvis. Imaging demonstrated growth of the parauterine lesion over three months. Due to symptoms and progression on imaging three months after recurrence, she restarted PLD and received 11 cycles with a total dose of 462 mg/m2, for a total cumulative PLD dose of 804 mg/m2. Five months later, imaging demonstrated that the DTF had not progressed and appeared to show some evidence of response with some decrease in enhancement of the tumor. One year after stopping the second series of PLD and 32 months after starting the series, imaging showed no change in tumor size, but some enhancement persisted.

The left ventricular ejection fraction (LVEF) by multigated acquisition (MUGA) scan was 48% when PLD was first begun, 43% one year later, and 50% with normal left ventricular (LV) size and function 15 months after starting PLD; no cardiac symptoms were noted. When PLD was reinstituted six years after completion of the first course of PLD, echo showed an ejection fraction (EF) of 55-60% with normal LV size and function. Echo three months after completing the second series of PLD showed an EF of 50-55% with normal LF systolic function.

Case 2 

**Figure 4:**
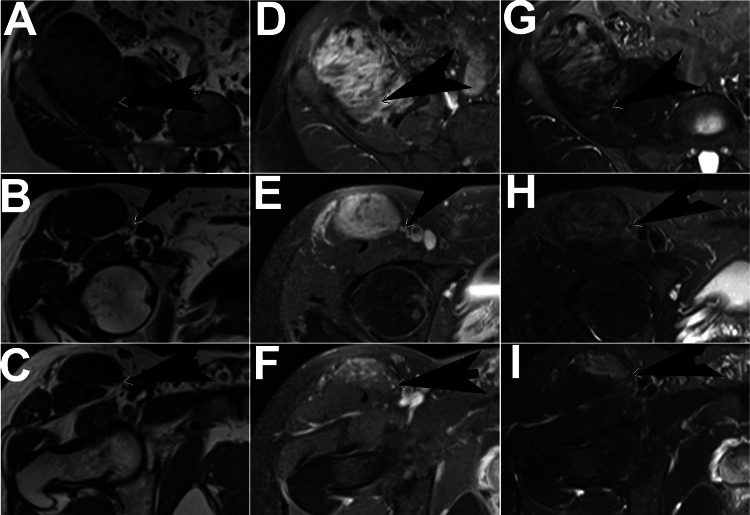
Sequential axial MR images of Case 2 Left column (A-C): T1 sequence; middle column (D-F): T1 fat saturation sequence post contrast; right column (G-I): T2 fat saturation. Black arrows indicate nerves: in the top row, the femoral cutaneous nerve (A, D, G) and in the second (B, E, H) and third rows (C, F, I), the femoral nerve

**Figure 5:**
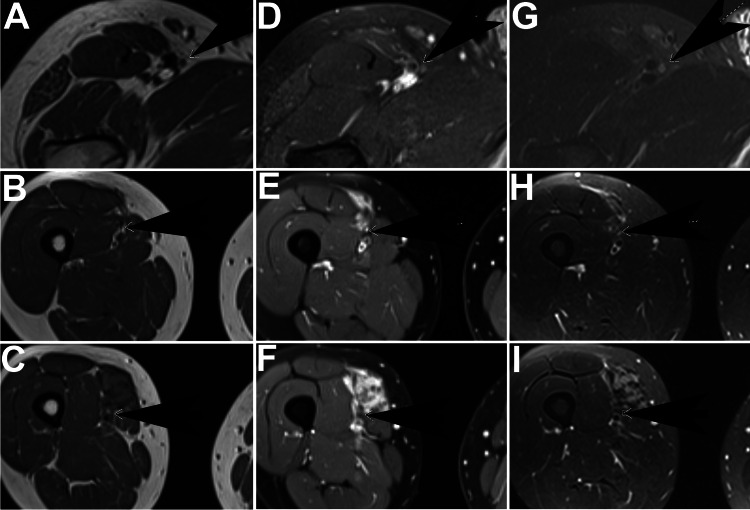
Sequential axial MR images of Case 2, continuation from Figure 4 Left column (A-C): T1 sequence; middle column (D-F): T1 fat saturation sequence post contrast; right column (G-I): T2 fat saturation. Black arrows indicate the saphenous nerve

A 38-year-old white man presented with gradually increasing pain and a mass in the right thigh. The family history was unremarkable, and there was no history of colon polyps or colon cancer. Physical examination revealed a nontender firm right anterior thigh mass and fullness in the right lower quadrant. A 3-cm lipoma in the upper back was also noted. MRI showed the thigh mass and what appeared to be a distinct large intra-pelvic mass of approximately 20×8 cm. Both lesions had T1 and T2 signals (Figure 4 and Figure 5). 

No association between the two lesions was found in multiple reviews, and they were felt to not be contiguous. Biopsy of both the thigh mass and the pelvic mass revealed DTF with nuclear beta-catenin staining (Figure 1). Both tumors had a CTNNB1 S45F mutation, which may be associated with a more aggressive phenotype. A PET-CT scan demonstrated the DTF lesions and also a PET-positive nodule in the rectum, as well as two liver lesions felt to be hemangiomas. Colonoscopy and polypectomy revealed a 4-mm well-differentiated adenocarcinoma in a tubulovillous adenoma with high-grade dysplasia with a normal pattern of mismatch repair protein expression. The rectal tumor did not have a CTNNB1 mutation; it was also normal for mismatch repair genes and showed no alterations of the amino acid sequence of BRAF, KRAS, or NRAS. A sessile serrated adenoma was found in the right ascending colon, and a tubular adenoma and sessile serrated adenoma, both negative for high-grade dysplasia, were noted in the hepatic flexure. No CTNNB1 was found in the colon polyp, thus excluding a germline mutation or chimera.

Analysis with the ColoNext panel from Ambry Genetics (Aliso Viejo, California, United States) was done as part of a cancer genetics risk evaluation and revealed a variant of unknown significance (VUS) in the MSH6 gene, p.P1082L. This is a missense mutation. No mutations were found in any of the other tested genes: APC, BMPR1A, CDH1, CHEK2, EPCAM, GREM1, MLH1, MSH2, MUTYH, PMS2, POLD1, POLE, PTEN, SMAD4, STK11, and TP53.

Due to a slight increase in tumor size and mild increase in symptoms, treatment with PLD was begun nine months after diagnosis, initially at 40 mg/m2 every four weeks. Symptoms improved, and seven months later, imaging showed a response. After 10 monthly cycles, cycle length was increased with continued symptomatic response and gradual tumor shrinkage. He received a total of 18 cycles over 21 months. Imaging two years after the last PLD and 3.7 years after initiation of treatment showed the response was maintained with a stable tumor size as compared with 14 months before (24 months after the last PLD), with decreased T2 hyperintensity and decreased enhancement of the tumors as compared with nine months earlier; he was asymptomatic. His aggressive fibromatosis was initially felt to be involved with two separate tumors, one in the right hip and one in the right thigh, both with S45F mutation. After later review, it was thought that these tumors were likely continuous, with evidence of subtle tumor tracking along nerves connecting the two main tumors (Figure 4 and Figure 5).

Case 3

**Figure 6:**
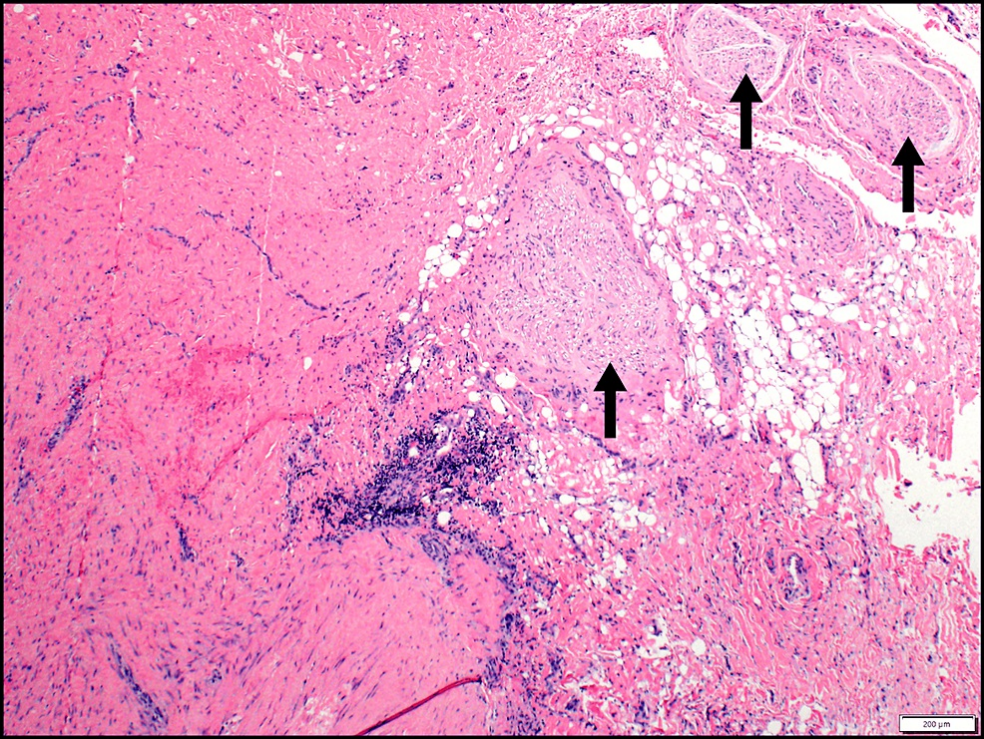
DTF resected from the left neck of Case 3 Deep DTF (left) resected from the left neck of Case 3, infiltrating the adipose tissue and in close association with large nerve bundles (arrows) (H&E ×40) DTF: desmoid-type fibromatosis; H&E: hematoxylin and eosin

A 60-year-old woman developed a nodule near the left clavicle that grew significantly over three months and was associated with some tingling in the arm. She had a history of hypertension, depression, migraines, gastroesophageal reflux disease (GERD), obstructive sleep apnea (OSA), and hypothyroidism; the family history was unremarkable. Biopsy showed DTF, and eight months after presentation, she had a subtotal surgical resection of a 2.8x3.3x6.9 cm mass involving the scalene muscles and the brachial plexus (Figure 6).

**Figure 7:**
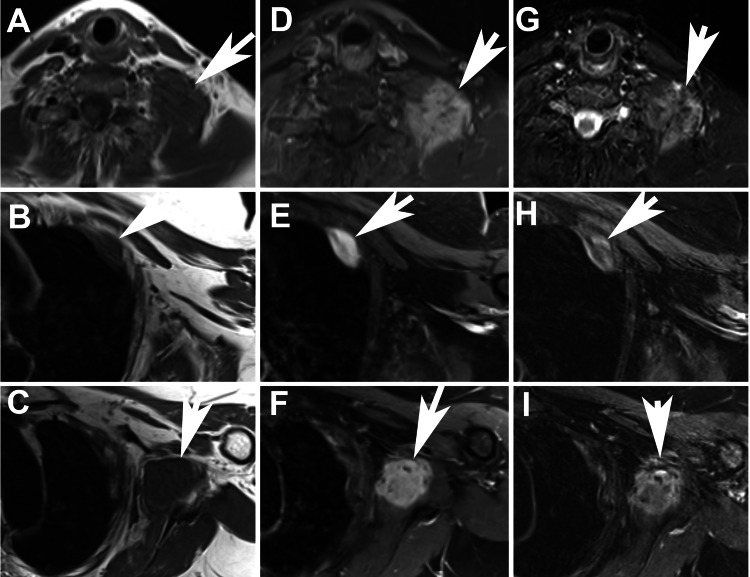
Axial MR images of Case 3 Axial MR images of Case 3. Top row (A, D, G): neck lesion; middle row (B, E, H): chest wall lesion; bottom row (C, F, I), subscapularis lesion. Left column (A-C): T1 sequence; middle column (D-F): T1 fat saturation sequence, post contrast; right column (G-H): STIR sequence. Arrows indicate tumor STIR: short tau inversion recovery

A chyle leak developed, and a thoracoscopic thoracic duct ligation was performed four days later. She completed radiation therapy three months later but developed a recurrence of tingling in the left arm three months after completing radiation. A recurrent tumor was noted, and she started methotrexate and vinblastine; due to toxicity, this was changed to every two-week treatment. The pain increased, and due to disease progression, she started PLD five months after starting methotrexate/vinblastine. PLD was given monthly for 13 months initially at 45 mg/m2 with a subsequent gradual reduction to 29 mg/m2 at 40-50-day intervals with gradual improvement in symptoms and a total dose of 450 mg/m2. Imaging showed a decrease in tumor size but persistent tumor. She developed pneumonia about three years after the last PLD, and imaging showed an asymptomatic chest wall tumor that was new from five years earlier. Further imaging showed a second tumor in the left axilla (Figure 7).

Biopsy of both lesions revealed DTF. Imaging of the original DTF lesion showed stable disease (Figure 7). Four months later, a review of the new CT images showed no change in the two new DTF lesions. The original DTF lesion in the neck was slightly smaller than at the time of the last PLD, having decreased from ~4.5x5.6x2.4 cm to ~1.2x3.5x3.1 cm. Image review showed that none of the three DTF lesions (neck, axillary, and chest wall) clearly communicated with the others. Each of the three separate DTF lesions had an S45F mutation in CTNNB1. Separate testing of a benign region in the primary resection specimen showed no germline beta-catenin mutation in the area of normal tissue near the DTF lesion. Imaging 5.4 years after the last PLD showed no progression of the DTF lesions and a slight decrease in the size of the left chest wall lesion.

## Discussion

While multiple DTFs are known to occur in some patients with germline APC mutations, they are very rare in sporadic DTF. Multiple DTFs should lead to a thorough evaluation of the colon and testing for APC mutation. While multifocal sporadic DTF has been reported, almost all reported cases have been regional [7-11]. A retrospective review of all cases between 1982 and 1991 at Memorial Sloan Kettering Cancer Center (MSKCC) found 124 patients with desmoid tumors, six of which were multifocal and not associated with FAP; in each case, the tumors were restricted to one anatomic location [8]. We report three cases of DTF that were initially thought to represent multifocal sporadic disease. These are the only three cases we have seen in our soft tissue tumor program in the last 30 years that were considered to be multifocal diseases in the absence of a germline APC mutation.

We now think that two of these cases reflect "subclinical" disease spread along nerves, suggesting they reflected a single atypical extensive tumor rather than true multifocal disease. The neural spread of DTF is reminiscent of NMC, a peripheral nerve malformation. NMC is frequently associated with aggressive fibromatosis (NMC-DTF), a rare disease of unclear pathogenesis that also frequently bears a CTNNB1 mutation [14-16,18,19]. This raises the question of whether DTF spread along nerves could appear as distinct multifocal lesions while actually being contiguous. Interestingly, on close questioning, Case 1 felt that as a child she had difficulty sitting cross-legged without discomfort. It is possible she had an NMC that was subclinical, and the lesion was only recognized subsequently in the form of a large DTF. Case 3 was considered to represent true multifocal tumor development, possibly due to tumor seeding at the time of chest surgery.

NMC is a developmental lesion in which mature skeletal muscle cells, or rarely smooth muscle cells, infiltrate and enlarge peripheral nerves, typically of large nerves or plexuses, but also cranial nerves [14-16,18-23]. NMC is quite rare, and in some cases, DTF arises in the NMC lesion (NMC-DTF). In one study, three cases of NMC and NMC-DTF each had an identical CTNNB1 mutation [18,19]. In one report of seven NMC-DTF cases, six had an S45X mutation (five S45F and one S45P), and one had a T41A mutation [18]. In a report of five cases of NMC, three had a S45F mutation, and two T41A [14]. In general, ~30% of sporadic DTF cases have a T41A mutation [6,24-26]. Thus, the high frequency of S45X mutations reported in NMC-DTF is noteworthy. Our two cases in which the DTF tracked along nerves both had an S45F mutation. Given that one report of three patients found identical CTNNB1 mutations in the NMC lesion and also the associated NMC-DTF, Carter et al. have suggested that a CTNNB1-mutated myofibroblast precursor can be induced to proliferate by a trigger, similar to typical DTF [3,16,18,19,27,28]. One study described ultrasound characteristics of NMC in seven patients [28]; CTNNB1 mutation was reported in one as S45F [28]. MRI characteristics of NMC have been described [29], and observation rather than biopsy of potential NMC has been suggested [18,19,28,30].

Maldonado et al. described eight cases of DTF, three of which were multifocal, associated with NMC [16]. In these cases, the DTFs arose only within the NMC-affected nerve territory. CTNNB1 mutations were not reported in these patients. These cases are remarkably like our Case 1 in that the lesions followed nerves. Gwynne-Jones et al. reported a case like our Case 1 who presented with a tumor in the L knee at age 26 [31]. This was removed, but two years later at age 28, a recurrence developed in the mid-thigh, which was removed. Another recurrence developed distally and was removed shortly thereafter. At age 33, a tumor developed in the L gluteus maximus and was biopsied; she was treated with tamoxifen, and the tumor was stable for 14 years.

Shimoyama et al. [32] described a case of a DTF in the right popliteal fossa that was resected, and three years later, a new DTF appeared in the left popliteal fossa. One year after resection of the second tumor, another DTF recurred in the left popliteal fossa and was partially excised due to attachment to the peroneal nerve. Six years later, she had a left femoral neck fracture, and 1.5 years later, she developed a DTF in the posterior left thigh, which was excised. A year later, a recurrent DTF developed that surrounded the sciatic nerve; an intra-lesional excision was performed, but the tumor recurred quickly and was treated with radiation therapy with stabilization. This case is also very similar to our Case 1 and could possibly reflect subclinical disease extension along nerves. Of note, like our case, she had hypothyroidism.

Case 3 represents a regional sporadic multifocal DTF not clearly associated with a nerve. Genetic analysis demonstrated the same S45F CTNNB1 mutation in each tumor. The probability of developing three spontaneous identical CTNNB1 mutations is very low. Analysis of adjacent normal fibroblasts revealed no detectable mutation as might be seen in a chimera. It remains possible that an undetected genetic variation could lead to an increased incidence of CTNNB1 mutation in this patient. Germline APC mutations that result in FAP have an increased incidence of DTF, typically in the abdomen especially the mesentery, and the location of the APC mutation affects the clinical expression of the disease. In mice, breeding APC-deficient mice in a p53-deficient background markedly increases multifocal DTF development [33]. The results suggest either a form of regional metastasis or a unique susceptibility to CTNNB1 mutation in this region of the patient. It is possible that the previous surgery in the chest cavity contributed to the local spread of tumor cells in our case. Bauer et al. described two patients with multiple DTFs occurring after surgical resection [34]. These are reminiscent of our Case 3.

Bekers et al. described six cases of multifocal DTF, four of which were locoregional [35]. Of those, two had a T41A mutation, and two had no CTNNB1 mutation identified. Two non-locoregional DTFs had an S45P and a T41A CTNNB1 mutation, respectively. These are more like our Case 3. Doyen et al. reported two patients with more than one DTF lesion [36]. In one case with four DTF lesions (two mesenteric and two abdominal walls), two lesions had no mutation, one had T41A, and the other had both S45P and S45Y CTNNB1 mutations. In the other case, one paravertebral DTF lesion expressed an S45P, and the other mesenteric DTF harbored a T41A CTNNB1 mutation. In Case 1, there was a one-year interval between the first and subsequent three lesions, while in Case 2, there was a one-year interval between the two lesions.

Taken together, our cases and cases presented in the literature confirm the very rare occurrence of multifocal sporadic DTF and also suggest that some cases of apparent multifocal DTF may represent subclinical spread along nerves, like that seen in the rare syndrome of NMC-DTF. It is interesting to speculate that the growth factor milieu of peripheral nerves could push DTF precursors to exhibit morphology more like the skeletal muscle than that seen in DTF not associated with NMC. Our two cases 1 and 2 may represent unrecognized NMC-DTF or DTF growing along nerves in response to an unidentified growth factor. Interestingly, the transcription factor SOX11, which accelerates in vivo nerve regeneration, was found to be over-expressed in DTF [2,37]. Another possibility is that a somatic CTTNB1 mutation occurring early in development might result in the entrapment of the mutated progenitor cell along the path of a growing nerve, with a subsequent stimulus inducing the formation of DTF. All three of our cases had an S45F CTNNB1 mutation. Several studies have reported an association of recurrence risk and CTNNB1 mutation status with S45F increasing recurrence risk after surgery [26,38,39] though not observed in all studies [40,41]. The results also provide further data supporting the low cardiotoxicity of long-term PLD use.

## Conclusions

This report and cases presented in the literature confirm the very rare occurrence of multifocal sporadic DTF and also suggest that some cases of apparent multifocal DTF may represent subclinical spread along nerves, like that seen in the rare syndrome of NMC-DTF. It is possible that the growth factor milieu of peripheral nerves could push DTF precursors to exhibit morphology more like the skeletal muscle than that seen in DTF not associated with NMC. Further genetic studies of DTF cases may be useful in predicting clinical behavior. The results also provide further data supporting the low cardiotoxicity of long-term PLD use.

## References

[1] (2020). The management of desmoid tumours: A joint global consensus-based guideline approach for adult and paediatric patients. Eur J Cancer.

[2] Misemer BS, Skubitz AP, Carlos Manivel J (2014). Expression of FAP, ADAM12, WISP1, and SOX11 is heterogeneous in aggressive fibromatosis and spatially relates to the histologic features of tumor activity. Cancer Med.

[3] Skubitz KM (2017). Biology and treatment of aggressive fibromatosis or desmoid tumor. Mayo Clin Proc.

[4] MacFarlane J (1832). Clinical reports of the surgical practice of the Glasgow Royal Infirmary. Clinical reports of the surgical practice of the Glasgow Royal Infirmary.

[5] Sanders R, Bennett M, Walton JN (1983). A multifocal extra-abdominal desmoid tumour. Br J Plast Surg.

[6] Le Guellec S, Soubeyran I, Rochaix P, Filleron T, Neuville A, Hostein I, Coindre JM (2012). CTNNB1 mutation analysis is a useful tool for the diagnosis of desmoid tumors: a study of 260 desmoid tumors and 191 potential morphologic mimics. Mod Pathol.

[7] Maurer F, Horst F, Pfannenberg C, Wehrmann M (1996). Multifocal extra-abdominal desmoid tumor--diagnostic and therapeutic problems. Arch Orthop Trauma Surg.

[8] Fong Y, Rosen PP, Brennan MF (1993). Multifocal desmoids. Surgery.

[9] Goy BW, Lee SP, Eilber F (1997). The role of adjuvant radiotherapy in the treatment of resectable desmoid tumors. Int J Radiat Oncol Biol Phys.

[10] Grünhagen DJ, de Wilt JH, Verhoef C, van Geel AN, Eggermont AM (2005). TNF-based isolated limb perfusion in unresectable extremity desmoid tumours. Eur J Surg Oncol.

[11] Rock MG, Pritchard DJ, Reiman HM, Soule EH, Brewster RC (1984). Extra-abdominal desmoid tumors. J Bone Joint Surg Am.

[12] Lewis JJ, Boland PJ, Leung DH, Woodruff JM, Brennan MF (1999). The enigma of desmoid tumors. Ann Surg.

[13] Bonvalot S, Desai A, Coppola S, Le Péchoux C, Terrier P, Dômont J, Le Cesne A (2012). The treatment of desmoid tumors: a stepwise clinical approach. Ann Oncol.

[14] Brandao IC, de Souza FS, de Amoreira Gepp R (2021). Neuromuscular choristoma: report of five cases with CTNNB1 sequencing. J Neuropathol Exp Neurol.

[15] Maldonado AA, Broski SM, Carter JM, Spinner RJ (2023). Unrecognized neuromuscular choristoma with recurrent desmoid-type fibromatosis and Marjolin ulcer: expanding the spectrum of neuromuscular choristoma sequelae within the nerve territory? Illustrative case. J Neurosurg Case Lessons.

[16] Maldonado AA, Spinner RJ, Broski SM, Stone JJ, Howe BM, Carter JM (2020). Neuromuscular choristoma-associated desmoid-type fibromatosis: establishing a nerve territory concept. Acta Neurochir (Wien).

[17] Skubitz KM, Manivel JC, Clohisy DR, Frolich JW (2009). Response of imatinib-resistant extra-abdominal aggressive fibromatosis to sunitinib: case report and review of the literature on response to tyrosine kinase inhibitors. Cancer Chemother Pharmacol.

[18] Carter JM, Howe BM, Hawse JR, Giannini C, Spinner RJ, Fritchie KJ (2016). CTNNB1 mutations and estrogen receptor expression in neuromuscular choristoma and its associated fibromatosis. Am J Surg Pathol.

[19] Carter JM, Maldonado AA, Howe BM, Okuno S, Spinner RJ (2021). Frequent CTNNB1 p.S45 mutations and aggressive clinical behavior in neuromuscular choristoma-associated fibromatosis. Neurosurgery.

[20] Bonneau R, Brochu P (1983). Neuromuscular choristoma. A clinicopathologic study of two cases. Am J Surg Pathol.

[21] Kumar R, Howe BM, Amrami KK, Spinner RJ (2014). Neuromuscular choristoma of the sciatic nerve and lumbosacral plexus: an association with nerve-territory undergrowth in the pelvis affecting soft tissue and bone. Acta Neurochir (Wien).

[22] Van Dorpe J, Sciot R, De Vos R, Uyttebroeck A, Stas M, Van Damme B (1997). Neuromuscular choristoma (hamartoma) with smooth and striated muscle component: case report with immunohistochemical and ultrastructural analysis. Am J Surg Pathol.

[23] Zhao W, Zhu X (2022). A case of esophageal neuromuscular choristoma. BMC Gastroenterol.

[24] Alman BA, Li C, Pajerski ME, Diaz-Cano S, Wolfe HJ (1997). Increased beta-catenin protein and somatic APC mutations in sporadic aggressive fibromatoses (desmoid tumors). Am J Pathol.

[25] Crago AM, Chmielecki J, Rosenberg M (2015). Near universal detection of alterations in CTNNB1 and Wnt pathway regulators in desmoid-type fibromatosis by whole-exome sequencing and genomic analysis. Genes Chromosomes Cancer.

[26] Lazar AJ, Tuvin D, Hajibashi S (2008). Specific mutations in the beta-catenin gene (CTNNB1) correlate with local recurrence in sporadic desmoid tumors. Am J Pathol.

[27] Skubitz KM, Murugan P, Corless CL (2022). Biclonal desmoid-type fibromatosis with two beta-catenin mutations: evidence for the recruitment of normal myofibroblasts. Cureus.

[28] Guo W, Wang H, Chen T, Yang W, Wang SF, Chen SL (2022). Clinical features and ultrasound findings of a rare musculoskeletal system disease-neuromuscular choristoma. BMC Musculoskelet Disord.

[29] Niederhauser BD, Spinner RJ, Jentoft ME, Everist BM, Matsumoto JM, Amrami KK (2013). Neuromuscular choristoma: characteristic magnetic resonance imaging findings and association with post-biopsy fibromatosis. Skeletal Radiol.

[30] Hébert-Blouin MN, Amrami KK, Spinner RJ (2013). Addendum: evidence supports a "no-touch" approach to neuromuscular choristoma. J Neurosurg.

[31] Gwynne-Jones DP, Theis JC, Jeffery AK, Hung NA (2005). Long-term follow-up of a recurrent multifocal desmoid tumour treated with tamoxifen: a case report. J Orthop Surg (Hong Kong).

[32] Shimoyama T, Hiraoka K, Shoda T, Hamada T, Fukushima N, Nagata K (2010). Multicentric extra-abdominal desmoid tumors arising in bilateral lower limbs. Rare Tumors.

[33] Smits R, van der Houven van Oordt W, Luz A (1998). Apc1638N: a mouse model for familial adenomatous polyposis-associated desmoid tumors and cutaneous cysts. Gastroenterology.

[34] Bauer BM, Williams NL, Zuckerman LM (2019). Development of multifocal extra-abdominal desmoid fibromatosis after surgical resection. Clin Case Rep.

[35] Bekers EM, van Broekhoven DL, van Dalen T (2018). Multifocal occurrence of extra-abdominal desmoid type fibromatosis - a rare manifestation. A clinicopathological study of 6 sporadic cases and 1 hereditary case. Ann Diagn Pathol.

[36] Doyen J, Duranton-Tanneur V, Hostein I (2016). Spatio-temporal genetic heterogeneity of CTNNB1 mutations in sporadic desmoid type fibromatosis lesions. Virchows Arch.

[37] Skubitz KM, Skubitz AP (2004). Gene expression in aggressive fibromatosis. J Lab Clin Med.

[38] Colombo C, Miceli R, Lazar AJ (2013). CTNNB1 45F mutation is a molecular prognosticator of increased postoperative primary desmoid tumor recurrence: an independent, multicenter validation study. Cancer.

[39] van Broekhoven DL, Verhoef C, Grünhagen DJ (2015). Prognostic value of CTNNB1 gene mutation in primary sporadic aggressive fibromatosis. Ann Surg Oncol.

[40] Dômont J, Salas S, Lacroix L (2010). High frequency of beta-catenin heterozygous mutations in extra-abdominal fibromatosis: a potential molecular tool for disease management. Br J Cancer.

[41] Mullen JT, DeLaney TF, Rosenberg AE (2013). β-Catenin mutation status and outcomes in sporadic desmoid tumors. Oncologist.

